# Rare Comorbidity of Sickle Cell Disease and Wilms' Tumor: A Case Report

**DOI:** 10.1155/carm/2454588

**Published:** 2025-09-19

**Authors:** Philippe Masidi, Josué Lumbu, Henoch C. Kabeya, Aymar Akilimali, Mariana Kruger

**Affiliations:** ^1^Department of Pediatrics, University Clinics of Kinshasa, Faculty of Medicine, University of Kinshasa, Kinshasa, Democratic Republic of the Congo; ^2^Department of Biomedical Sciences, Faculty of Medicine, University of Kinshasa, Kinshasa, Democratic Republic of the Congo; ^3^Plastics and Human Health, Minderoo Foundation, Perth, Western Australia, Australia; ^4^Department of Research, Medical Research Circle (MedReC), Goma, Democratic Republic of the Congo; ^5^Department of Pediatrics and Child Health, Faculty of Medicine and Health Sciences, Stellenbosch University, Stellenbosch, South Africa

**Keywords:** malignancy, sickle cell anemia, Wilms tumor

## Abstract

Wilms' tumor (nephroblastoma) is among the most common childhood cancers in the Democratic Republic of Congo and other African countries. However, its association with sickle cell disease is rarely reported in the literature. We present a case of nephroblastoma associated with sickle cell disease. The management of such a case requires a multidisciplinary team in a specialized center. The combination of surgery with pre- and postoperative chemotherapy leads to excellent curative rates. Renal histology after nephrectomy provides the diagnosis and defines the tumor type. Multiple mutations leading to cancers in humans may explain the coexistence of these two genetic diseases with affected genes found in the same chromosome. Further research to provide a molecular genetic explanation of such comorbidity is recommended.

## 1. Introduction

Malignancies in individuals with sickle cell disease (SCD) have been sporadically reported over the past 5 decades, primarily through case reports and small case series [1–6]. A single-institution study estimated the incidence of cancer in SCD patients at 1.74 cases per 1000 patient-years [7]. However, comprehensive data on the prevalence and types of cancers in this population remain scarce. This gap in knowledge is particularly important given concerns regarding the potential oncogenic risk of hydroxycarbamide, a commonly used disease-modifying treatment for SCD. In the African pediatric population, common cancers include lymphomas, Wilms tumor (WT), Kaposi's sarcoma, and retinoblastoma [8]. Understanding the interplay between SCD and malignancy is essential for tailoring care strategies in this unique patient population.

WT usually appears in children under 5 years of age, but it can be diagnosed in older children and, exceptionally, in adulthood. The histology is distinct from that of adult kidney cancers, as WT arises from embryonal tissue [9]. The exact cause of WT is unknown, and to date, research has not found links with the environment, diet, lifestyle, or psychological factors. A minority (10%) of cases are associated with malformations linked to genetic abnormalities that can promote development. There are also familial forms with genetic transmission [10].

The coexistence of SCD and WT in a single child represents a complex clinical scenario. Both conditions independently have significant morbidity and mortality, and their overlap causes diagnostic and therapeutic challenges. Clinical features such as pain, anemia, or organomegaly may be mistakenly assigned to vaso-occlusive crises and splenic complications of SCD, potentially delaying recognition of an underlying tumor. In addition, the management of WT, involving surgery, chemotherapy, and sometimes radiotherapy, can be complicated by the fragile baseline health of SCD patients, who have susceptibility to infections, impaired organ function, and perioperative risks [11]. Optimizing treatment in such cases therefore requires a multidisciplinary approach, balancing curative oncologic strategies with meticulous supportive care for SCD. The objective of this report is to present a rare case of WT in a child with SCD and to underline the unique diagnostic and therapeutic challenges posed by this comorbidity.

## 2. Case Presentation

A nine-year-old boy with a known history of SCD presented with symptoms later attributed to a coexisting WT. He was successively followed at two separate medical centers, though the specifics of the medications administered were unclear to the family and yielded no significant improvement. He was the youngest of six siblings, with a family history of SCD, and had undergone four blood transfusions since birth. At presentation, he reported abdominal pain associated with defecation, persistent fever, and recurrent vaso-occlusive crises. Additionally, he experienced osteoarticular pain, further complicating his clinical picture.

On admission, he weighed 18 kg with a height of 120 cm (below the third percentile). He was feverish. He was pale with subicteric sclera. His heart rate was 140 beats per minute with signs of pericardial friction rub. The respiratory examination was marked by tachypnea at 62 breaths per minute, with dullness and decreased fremitus over the lower two-thirds of the left posterior pulmonary field. His oxygen saturation was 89% on room air. On abdominal examination, he had a distended abdomen, with portosystemic collateral pathways present. There was a firm, painful mass, extending from the right hypochondrium to the ipsilateral iliac fossa (approximately 15 cm in vertical diameter) and crossing the midline into the left hypochondrium on the mid-clavicular line (approximately 20 cm in horizontal diameter). The child also presented with scalp pain and swelling.

**Figure 1:**
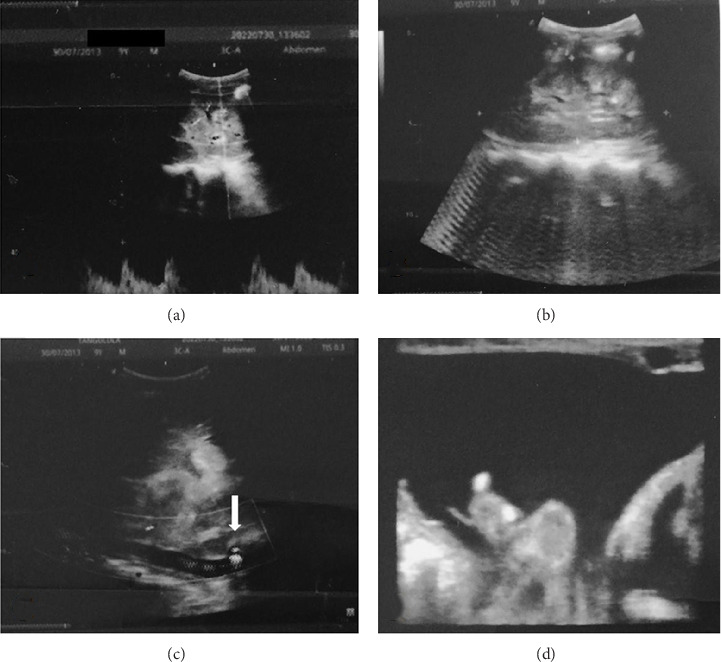
Abdominal ultrasonography features. (a, b) Enlarged right kidney in form of a hyper- and hypo-echogenic mass with microcalcifications. (c) Thrombotic, stenosing infiltration of infra and retro hepatic segments of the vena cava. (d) Free ascites of medium abundance.

Both urine and biochemical investigations were unremarkable (serum glucose, creatinine, urea, and liver enzymes). There was a normochromic normocytic anemia and hyperleukocytosis with neutrophilic predominance on full blood count. He had an elevated serum CRP. The abdominal ultrasound showed the presence of a large solid right renal mass with extension outside the kidney and the classic spur sign. The renal volume was 175.8 × 159.8 × 189.2 mm^3^, suggestive of a right unilateral renal tumor with local extension, complicated by venous thrombosis in the renal vein and inferior vena cava, sparing the suprahepatic segment. There were also diffuse ascites, celiac lymphadenopathy, subpleural pulmonary nodules, and calcified micronodular splenic hypotrophy, with mass effect on neighboring organs. There were no liver metastases or portal thrombosis (Figure 1).

Considering the epidemiological, clinical, and ultrasonographic findings, suspected higher-stage WT was diagnosed.

In clinically stable patients without contraindications, nephrectomy prior to chemotherapy is recommended. In this case, however, the clinical instability and thrombotic extension into the inferior vena cava made upfront surgery high risk. Therefore, neoadjuvant chemotherapy was indicated to achieve tumor shrinkage and minimize surgical risk. Accordingly, a 24-week course of vincristine, dactinomycin, and doxorubicin was started, as well as antibiotics for suspected sepsis, namely, ceftriaxone and amikacin. Pain was managed with paracetamol and tramadol. Further investigations were required including blood culture and urine microscopy, culture, and sensitivity. The patient was clinically stabilized after 5 days of treatment. However, on Day 7, the clinical course was quickly complicated by respiratory distress and fever, which after clinical examination was suspected to be secondary to bronchopneumonia. He suddenly developed a cardiopulmonary arrest for which he underwent resuscitation for 45 min. However, despite these efforts, the patient died during resuscitation.

**Figure 2:**
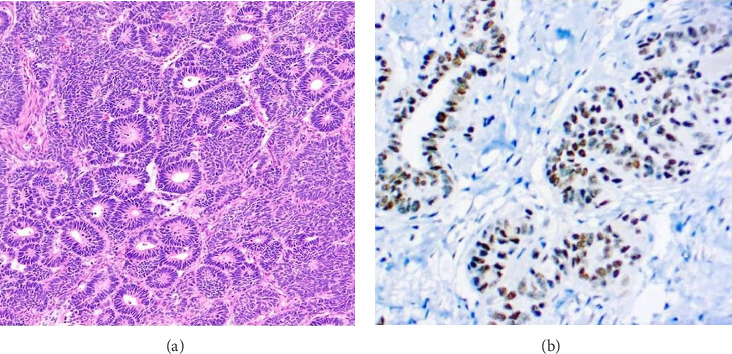
Microscopic examination. (a) Histological features of a triphasic WT pattern with abundance of epithelial component and nodules of blastema with a scarce part of fibroblastic stroma. (b) Immunohistochemistry revealing positive WT1 IgG antibody in tumoral tissues.

A postmortem kidney histopathological examination was performed to confirm the diagnosis and classify the tumor after obtaining the deceased patient's family's consent. The examination revealed morphological features compatible with triphasic WT, and immunohistochemistry was positive for WT1, confirming the diagnosis of WT (Figure 2). The cause of death was not determined, as consent for a full postmortem examination was not granted by the family.

## 3. Discussion

This coexistence of SCD and WT in the same patient raises intriguing clinical but also genetic considerations. SCD most commonly stems from a point mutation in the β-globin gene, HBB (on chromosome 11p15.5), leading to the synthesis of structurally abnormal hemoglobin, HbS [12]. WT, on the other hand, has been linked to abnormalities in genes regulating renal and genitourinary development, most notably WT1 (11p13) and loci within 11p15.5, where imprinting defects and mutations lead to development of tumors. Although WT is one of the most common cancers in the local context, data on its association with SCD in the literature are lacking [13]. Schultz et al. reported only five cases with associated WT out of 16,613 SCD patients (0.0003%) [14]. Less globally reported in the literature, a clear explanation for this rare coexistence of SCD and WT is not available.

Currently, the accumulation of multiple mutations is well recognized to be a mechanism underlying malignancy transformation [15]. Although there is no published evidence supporting a predisposition to WT in a patient with HBB mutation, it is plausible that, in rare cases, the close proximity of the HBB gene and WT-related loci may reflect a shared susceptibility region on 11p15.5, on which genetic or epigenetic disruptions could contribute to the coexistence of SCD and WT [16]. Although this remains speculative, the concept of a mutational hotspot within chromosome 11p provides a biologically plausible explanation for such a comorbidity [17]. The strong association between WT and other syndromes, such as WT, aniridia, genitourinary anomalies, and intellectual disability (WAGR) and Beckwith–Wiedemann syndrome, secondary to genetic and epigenetic abnormalities in the same chromosomal region, suggests the need for genetic analyses [18]. However, without molecular testing, this hypothesis remains unproven.

Optimal management of children with WT and SCD requires early diagnosis, timely referral to specialized centers, and multidisciplinary coordination, including pediatric oncology, hematology, surgery, anesthesiology, and supportive care teams. Current guidelines, such as those from the Children's Oncology Group and the International Society of Pediatric Oncology, recommend standardized diagnostic imaging, histopathological confirmation, risk stratification, and multimodal treatment combining surgery, chemotherapy, and in some cases radiotherapy [19]. For patients with SCD, additional considerations include optimization of hemoglobin levels, transfusion protocols to reduce perioperative complications, and vigilant monitoring for vaso-occlusive crises or infectious events. However, in resource-limited settings, including many low-income countries, adherence to these standards remains challenging due to the unavailability of advanced imaging, limited access to chemotherapy agents, absence of molecular diagnostics, and shortage of trained specialists. Strengthening health systems through earlier case detection at the primary care level, establishment of strict referral pathways, implementation of adapted treatment protocols that consider drug availability, and the use of regional centers of excellence could substantially improve outcomes. Partnerships with international networks and capacity-building initiatives are also essential to narrow the gap between global recommendations and local practice, ultimately reducing preventable mortality in children facing this dual burden.

The prognosis of WT is favorable when it is diagnosed and treated early. Late diagnosis and/or late referral to a specialized center can worsen the prognosis of children with WT. The SCD-WT association requires multidisciplinary management. In developing countries, new therapeutic approaches for SCD are not applicable to several factors, such as the lack of human, material, and financial resources. On the other hand, the socioeconomic impact of cancer is remarkable in developing countries. The cost of treatment is significant, as oncology encounters several barriers to its emergence in sub-Saharan Africa.

This case report has several limitations, primarily related to the resource-limited setting in which the patient was managed. There was no access to advanced diagnostic technologies that could have facilitated early detection and more precise characterization of the disease. Finally, the cause of death could not be determined, as the patient's family did not provide consent for a full postmortem examination, leaving important questions about the rapid clinical deterioration unanswered.

## 4. Conclusion

This report illustrates a rare comorbidity of two diseases with the potential to worsen each other's prognosis. Such patients would benefit from multidisciplinary care. Early diagnosis and initiation of optimal treatment can improve the prognosis and reduce morbidity and mortality in these patients. Further investigations are recommended to provide a biomolecular explanation of these comorbidities.

## Data Availability

The data that support the findings of this study are available from the corresponding author upon reasonable request.
